# Supplementary material for: Sequential Defense Strategies: From Ant Recruitment to Leaf Toughness
Source: Plants (Basel). 2024 Dec 27;14(1):49. doi: 10.3390/plants14010049 (PMC11722744; doi:10.3390/plants14010049)
Supplement: Supplementary file 1 [file plants-14-00049-s001.zip › plants-3317498-supplementary.pdf]

Supplementary material

Plant defenses turn over and response after simulated herbivory

dos Santos, D. F. B.[1]; Calixto, E. S.[2]; Torezan-Silingardi, H. M.[3] & Del-Claro, K.[3]*

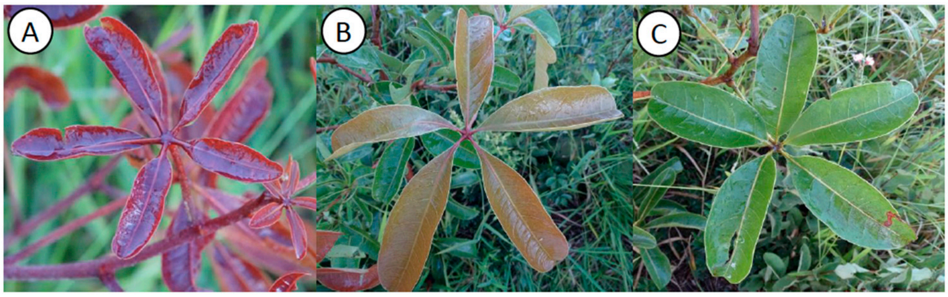

Figure S1. Foliar stages of throughout leaf development. The coloration of each stage was associated with leaf age. (A) young leaf (up to two weeks), (B) intermediate leaf (two to four weeks), and (C) adult (at least one month).

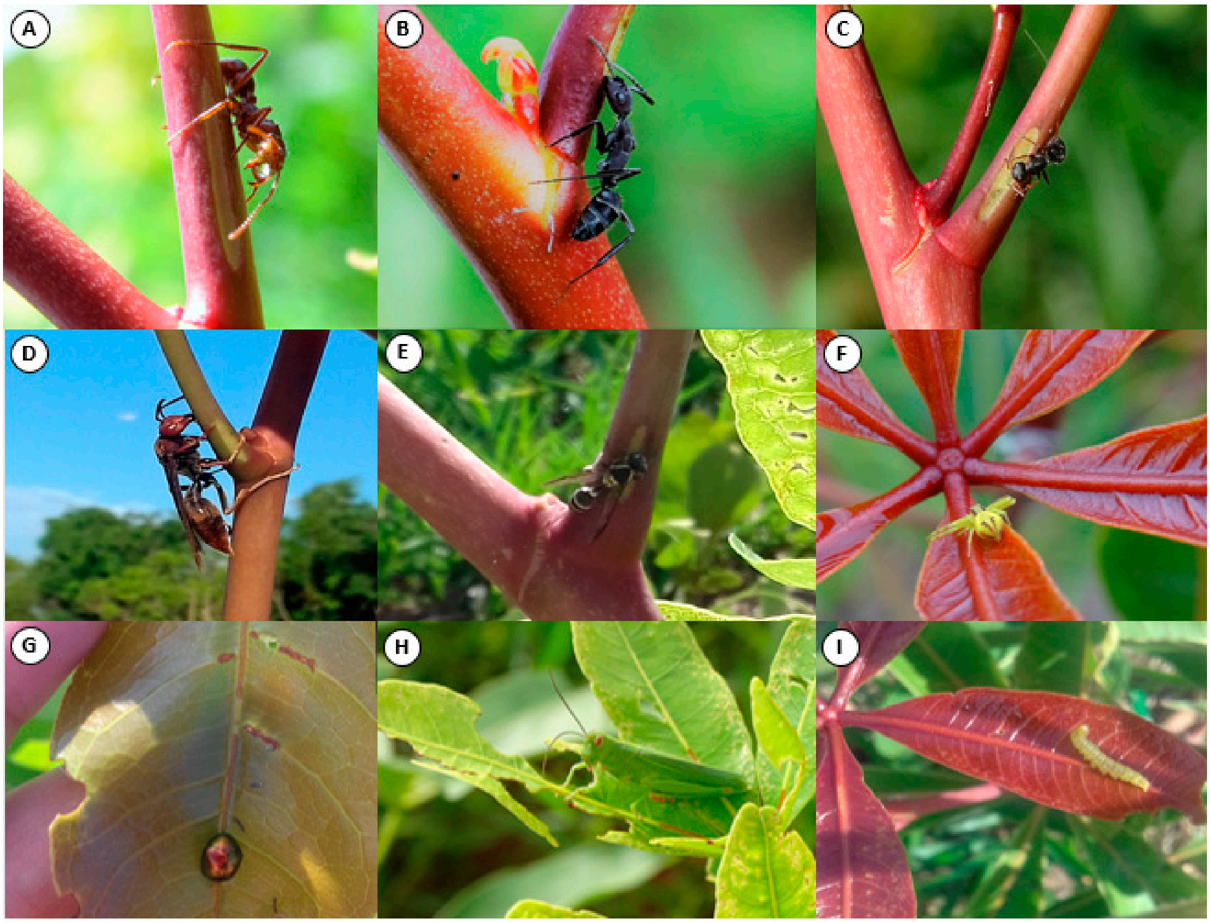

Figure S2. Examples of extrafloral nectary visitors (A-F) and foliar herbivores (G-I) in *Eriotheca gracilipes*: (A) *Ectatomma tuberculatum*, (B) *Camponotus leydigi*, (C) *Camponotus crassus*, (D) *Polistes* sp., (E) *Polybia occidentalis,* (F) *Misumenops* sp. (Thomisidae), (G) *Lamprosoma* sp., (H) Acrididae, and (I) *Cerconota* sp.

Table S1. Total abundance of ants on *Eriotheca gracilipes*, in an area of Cerrado, Brazil. Ab.a – absolute abundance; Re.a – Relative abundance (%).

Subfamily	Treatments		
Species	Control<br>Ab.a (Re.a%)	10%<br>Ab.a (Re.a%)	50%<br>Ab.a (Re.a%)
**Formicinae**			
Camponotus crassus Mayr, 1862	174(43.9)	214(34.8)	183(33.3)
Camponotus blandus (Smith, F., 1858)	65(16.4)	191(31.1)	81(14.7)
Camponotus leydigi Forel, 1886	9(2.2)	6(0.9)	2(0.3)
Camponotus trapeziceps Forel, 1908	6(1.5)	2(0.3)	2(0.3)
**Ectatomminae**			
Ectatomma tuberculatum (Olivier, 1792)	37(9.3)	33(5.3)	25(4.5)
**Myrmicinae**			
Crematogaster sp.	24(6.0)	42(6.8)	71(12.9)
Solenopsis sp.	2(0.5)	0	5(0.9)
**Dolichoderinae**			
Tapinoma sp.	75(18.9)	116(18.8)	172(31.3)
Dorymyrmex sp.	3(0.7)	8(1.3)	8(1.4)
**Ponerinae**			
Neoponera villosa (Fabricius, 1804)	1(0.2)	2(0.3)	0

Table S2. Total abundance of herbivores and other visitors in *Eriotheca gracilipes*, in an area of Cerrado, Brazil. Absolute abundance (Relative abundance %). Among the observed visitors only herbivores were included in the table.

Family/Subfamily	Treatments			
Genus/Species	Control<br>Ab.a(Re.a%)	10%<br>Ab.a(Re.a%)	50%<br>Ab.a(Re.a%)	No Ants<br>Ab.a(Re.a%)
**Acrididae**				
Acrididae sp. 1	1(3.4)	1(5.5)	0	2(10)
Acrididae sp. 2	0	1(5.5)	0	0
**Alydidae**				
Alydidae sp.	0	0	0	1(5)
**Cerambycidae**				
Cerambycidae sp.	0	1(5.5)	0	1(5)
**Cercopidae**				
Deois flavopicta Stall, 1954	0	0	1(2.3)	0
**Cicadidae**				
Cicadidae sp.	2(6.8)	0	1(2.3)	0
**Chrysomelidea**				
Crepidodera sp.	0	0	0	1(5)
Lamprosoma sp.	1(3.4)	4(22.2)	1(2.3)	3(15)
Megalostomis gazela Lacordaire ,1848	1(3.4)	0	0	0
**Coreidae**				
Amorbus sp.	0	0	1(2.3)	0
Coreidae sp. 1	1(3.4)	1(5.5)	4(9.3)	1(5)
Coreidae sp. 2	1(3.4)	0	0	0
*Leptoglossu*s *zonatus*	0	0	1(2.3)	0

**Curculionidae**				
Curculionidae sp. 1	14(48)	10(55,5)	34(79)	3(15)
Curculionidae sp. 2	1(3.4)	0	0	0
**Oecophoridae**				
Oecophoridae sp.	4(13.7)	0	0	7(35)
**Pentatomidae**				
Pentatomidae sp.	0	0	0	1(5)
**Proscopiidae**				
Proscopiidae sp.	1(3.4)	0	0	0
**Pyrgomorphidae**				
Pyrgomorphidae sp.	1(3.4)	0	0	0
**Rhopalidae**				
Rhopalidae sp.	1(3.4)	0	0	0
